# CP5 system, for simple and highly efficient protein purification with a C-terminal designed mini tag

**DOI:** 10.1371/journal.pone.0178246

**Published:** 2017-05-25

**Authors:** Hiroyuki Takeda, Wei Zhou, Kohki Kido, Ryoji Suno, Takahiro Iwasaki, Takuya Kobayashi, Tatsuya Sawasaki

**Affiliations:** 1 Proteo-Science Center, Ehime University, Matsuyama, Ehime, Japan; 2 Department of Medical Chemistry and Cell Biology, Kyoto University Graduate School of Medicine, Yoshida, Sakyo-ku, Kyoto, Japan; 3 AMED-CREST, Japan Agency for Medical Research and Development, Chiyoda-ku, Tokyo, Japan; National Taiwan University, TAIWAN

## Abstract

There are many strategies to purify recombinant proteins of interest, and affinity purification utilizing monoclonal antibody that targets a linear epitope sequence is one of the essential techniques used in current biochemistry and structural biology. Here we introduce a new protein purification system using a very short CP5 tag. First, we selected anti-dopamine receptor D1 (DRD1) rabbit monoclonal antibody clone Ra62 (Ra62 antibody) as capture antibody, and identified its minimal epitope sequence as a 5-amino-acid sequence at C-terminal of DRD1 (GQHPT-COOH, D1CE sequence). We found that single amino acid substitution in D1CE sequence (GQHVT-COOH) increased dissociation rate up to 10-fold, and named the designed epitope sequence CP5 tag. Using Ra62 antibody and 2 peptides with different affinity, we developed a new affinity protein purification method, CP5 system. Ra62 antibody quickly captures CP5-tagged target protein, and captured CP5-tagged protein was eluted by competing with higher affinity D1CE peptide. By taking the difference of the affinity between D1CE and CP5, sharp elution under mild condition was achieved. Using CP5 system, we successfully purified deubiquitinase CYLD and E3 ubiquitin ligase MARCH3, and detected their catalytic activity. As to G protein-coupled receptors (GPCRs), 9 out of 12 cell-free synthesized ones were purified, demonstrating its purification capability of integral membrane proteins. CP5 tagged CHRM2 expressed by baculovirus-insect cell was also successfully purified by CP5 system. CP5 system offers several distinct advantages in addition to its specificity and elution performance. CP5 tag is easy to construct and handle because of its short length, which has less effect on protein characters. Mild elution of CP5 system is particulaly suitable for preparing delicate proteins such as enzymes and membrane proteins. Our data demonstrate that CP5 system provides a new promising option in protein sample preparation with high yield, purity and activity for downstream applications in functional and structural analysis.

## Introduction

Protein purification with affinity tags is an essential technique for recombinant protein sample preparation in current biochemistry and structural biology [1,2]. Using genetic engineering and affinity purification tag system, it is very easy and efficient to obtain pure and functionally active protein of interest with reduced time and effort. Affinity purification tag technologies have been developed and improved over the past 20 years. Some are widely used around the world, such as immobilized metal-ion affinity chromatography (IMAC) with poly-histidine tag [3], folded domain tags such as glutathione-S-transferase (GST) [4] and maltose binding protein (MBP) [5], and short epitope tag represented by FLAG M1 purification [6]. Even today, reports on new tag systems for protein purification are being published [2,7,8]. In many cases, it is difficult to achieve complete purity with single affinity purification step, even though those affinity purification methods are powerful and efficient enough. For example, sample preparation for crystallography requires extremely high quantity, purity and concentration of target protein. For that, researchers usually have to repeat several chromatography steps sequentially based on different principles, including affinity purification, ion exchange chromatography and size exclusion chromatography. In such a case, “dual tagging” or “multi tags” (e.g., FLAG tag and His_10_ tag fused at the N- and C-terminus of the target protein, respectively) are often designed to help improve purity and reduce purification steps [2,9–12]. Considering the compatibility between tags and target proteins, new affinity purification systems with high efficiency and versatility are still highly needed.

Affinity purification systems using the interaction between monoclonal antibody and the determinant epitope of the corresponding antigen have many advantages such as selectivity and stability in capture. The smallness of the tag sequence is another advantage, which has much less influence on the structure of target proteins. One possible problem of antibody-based purification is the elution efficacy. Commonly, target protein captured by anti-tag antibody is competitively eluted by high concentration of epitope peptide. Sometimes, however, competitive elution does not work well because of the very high affinity of capture antibody, which results in increased elution volume or low recovery. In such a case, more severe conditions, such as acid, alkaline, or high concentration of di-cation, are required to overcome the problem, which in turn of course, can be detrimental to protein activity and folding. Among many well-used systems, FLAG M1 purification shows excellent performance in elution. FLAG M1 antibody binds to FLAG tag at the N- terminus of the target protein in Ca^2+^ ion-dependent manner, and the binding can be easily and efficiently eluted by EDTA treatment [6,13]. FLAG M1 purification is often used for crystallography sample preparation of GPCRs, which are known for being unstable and difficult to handle integral membrane proteins [9,14–16].

Previously we reported AGIA tag system, which makes use of a high-affinity rabbit monoclonal antibody against dopamine receptor D1 (DRD1) clone Ra48 (Ra48 antibody) and its epitope sequence [17]. AGIA tag is demonstrated to have high sensitivity, specificity and stability, and it shows excellent performance in detection and capture of target protein. To make AGIA tag applicable for purification, we attenuated affinity between Ra48 antibody and AGIA tag by introducing amino acid substitution into AGIA tag. Modified AGIA tag-based purification showed high purity, but sharp elution was not fully achieved. In this paper, we designed a new affinity tag-based purification system; CP5 system. The CP5 system consists of anti-DRD1 rabbit monoclonal antibody clone Ra62 (Ra62 antibody) and two kinds of very short peptide sequences, which bind to Ra62 antibody with low and high affinity, respectively. With these peptides, capture and elution of target protein were performed effectively. Here, we address the property of CP5 system and its performance in purification of soluble enzymes and integral membrane proteins.

## Materials and methods

### General

Oligo nucleotides were purchased from Thermo Fisher Scientific. Synthetic peptides were provided by Sigma-Aldrich. All reagents were purchased from Nakarai Tesque unless otherwise specified.

### Cell-free protein synthesis

The following procedures were previously described [18–21]: wheat germ cell-free soluble protein synthesis, wheat germ cell-free membrane protein synthesis, construction of plasmids for cell-free protein synthesis. Wheat germ cell-free expression kit was purchased from CellFree Sciences. pEU-based expression vectors were used for wheat germ cell-free protein synthesis [20]. Genes of interest were subcloned into expression vectors using Gateway Technology (Thermo Fisher Scientific) or Gibson Assembly (New England Biolabs). Insertion, deletion and amino acid substitution were performed using PrimeSTAR Mutagenesis Kit (Takara Bio). Human genes such as DRD1, CYLD, ADORA2A, AGTR1, GHSR, P2RY2, RHO, PTH1R, FZD7, MARCH3, GABBR1 were obtained from the Mammalian Gene Collection (MGC) full-length cDNA clone set [22]. GHRHR, MCHR1, MRGPRX1 are from Flexi cDNA library (Promega).

### Production and preparation of Ra62 antibody

cDNAs for heavy and light chains of Ra62 antibody [21] were subcloned into the pcDNA3.4 expression vector using PCR and In-Fusion Reaction (Clontech). Ra62 antibody was expressed using the Expi293F Expression System (Thermo Fisher Scientific), according to the manufacturer’s instruction. The antibody secreted in culture medium was purified by protein G Sepharose 4 Fast Flow (GE Healthcare), and then buffer was exchanged to PBS by PD-10 column (GE Healthcare). Antibody concentration was determined by extinction coefficient method [23] with NanoDrop spectrophotometer (Thermo Fisher Scientific). Purified antibody was frozen and stored at -30°C. Preparations of Ra62 antibody conjugated Sepharose (Ra62 antibody Sepharose) was conducted using NHS-activated Sepharose 4FF (GE Healthcare), according to the manufacturer’s instructions. Two mg of Ra62 antibody was immobilized on 1 mL of Sepharose. Ra62 antibody Sepharose was stored at 4°C.

### AlphaScreen

Binding assay between Ra62 antibody and DRD1 fragment was conducted by AlphaScreen. Biotin ligation site (bls)-SrtA-DRD1 fragment proteins [21] were synthesized using wheat germ cell-free synthesis system, in which biotin and BirA biotin ligase was added to biotinylate bls enzymatically [24]. All AlphaScreen reactions were conducted in an Optiplate 384 titer plate (PerkinElmer). 1 μL of cell-free synthesized biotin-SrtA-DRD1 fragment and 30 ng of Ra62 antibody were mixed in 15 μL AlphaScreen buffer containing 100 mM Tris-HCl (pH8.0), 0.01% Tween20, 100 mM NaCl and 1 mg/mL bovine serum albumin (BSA), and incubated at 26°C for 30 min. Subsequently, 10 μL detection mixture containing 0.1 μL streptavidin-conjugated AlphaScreen donor beads and 0.1 μL protein A-conjugated AlphaScreen acceptor beads in AlphaScreen buffer was added to the mixture. After incubation at 26°C for 1 h, AlphaScreen chemiluminescence signal was detected by Envision multilabel reader (PerminElmer).

Recognition of terminus carboxyl group of D1CE epitope by Ra62 antibody was validated using peptide. Biotin-TSQNGQHPT-COOH and biotin-TSQNGQHPT-NH_2_ were chemically synthesized by Sigma-Aldrich. In addition to Ra62 antibody, Ra48 antibody was applied as negative control. 2 nM of a peptide was mixed with 4 ng of an antibody, 0.1 μL streptavidin-conjugated AlphaScreen donor beads and 0.1 μL protein A-conjugated AlphaScreen acceptor beads in AlphaScreen buffer. After incubation at 26°C for 1 h, AlphaScreen signal was detected.

### Immunoblot analysis

For sample preparation for SDS-PAGE, soluble proteins were mixed with ×3 SDS-PAGE sample buffer and denatured by heating at 99°C for 5 min. Denaturation of membrane proteins and cell extracts was conducted at 37°C for 30 min. After separation by SDS-PAGE using e-PAGEL 5–20% gel (ATTO), proteins were transferred to an Immobilon-P membrane (Millipore) using EzFastBlot (ATTO). After blocking with 5% skim milk solubilized in Tris-buffered saline containing 0.01% Tween20 (TBST) for 1 h, the membrane was treated for 1 h by primary antibodies at the appropriate concentration. The membrane was washed 3 times with TBST, and treated with the appropriate secondary antibody, anti-rabbit IgG-HRP or anti-mouse IgG-HRP antibody (GE Healthcare). After TBST washing for three times, the antibody was visualized using Immunostar LD (Wako). Chemiluminescent signal was detected by ImageQuant LAS 4000 imager (GE Healthcare).

### Mammalian cell culture

HEK293T, HeLa, MCF7, and CHO cells were incubated at 37°C under 5% CO_2_ in Dulbecco’s Modified Eagle Medium (DMEM) (Nissui) with 10% fetal bovine serum (Sigma-Aldrich), 2mM L-glutamine (Thermo Fisher Scientific) and antibiotics (100units/ml penicillin and 100μg/ml streptomyscin) (Thermo Fisher Scientific). Huh7 cells were incubated at 37°C under 5% CO_2_ in DMEM-high glucose (Wako) with 10% fetal bovine serum, 1×MEM NEAA (Thermo Fisher Scientific), 1mM sodium pyruvate and antibiotics (100units/ml penicillin and 100μg/ml streptomyscin). NIH3T3 cells were incubated at 37°C under 5% CO_2_ in DMEM-high glucose with 10% calf serum (Thermo Fisher Scientific) and antibiotics (100 units/mL penicillin and 100 μg/mL streptomycin).

### Biacore assay

Biacore experiment was conducted on a Biacore X100 apparatus (GE Healthcare). HBS-EP+ (10 mM Hepes-NaOH (pH 7.4), 150 mM NaCl, 0.05% Tween 20, and 3 mM EDTA) was used as running buffer. The temperature of the flow cells was kept at 25°C during assays.

To determine affinity between the tags and Ra62 antibody, a DRD1 fragment or D1CE/CP5 tag was fused with FLAG-GST using Gibson assembly. The fusion proteins were synthesized by cell-free system and purified by glutathione-Sepharose 4B (GE Healthcare) and used as analyte. Concentration of purified protein was assayed by extinction coefficient [23] with NanoDrop spectrophotometer. Extinction coefficient of each protein was calculated by using ProParam (http://web.expasy.org/protparam/) [25]. SPR assay was performed by capture method. Protein G was immobilized on a CM5 sensor chip by amine coupling at 6,000 RU. In each cycle, Ra62 antibody was captured on a measuring cell at 200 RU. Then an analyte protein was injected at 30 μL/min for 120 sec. After 120 sec of dissociation time, the chip surface was regenerated by injecting protein G-regeneration solution containing 10 mM NaOH and 0.5 M NaSCN for 30 sec at 10 μL/min. Affinity parameter was calculated by using BiaEvaluation software.

For elution assay, Ra62 antibody was immobilized on the measuring cell of a CM5 sensor chip by amine coupling at 6,000 RU. Anti-DRD1 antibody clone Ra51, which recognizes extracellular loop of DRD1, was immobilized on the reference cell at same level as isotype control. The assay was conducted at 10 μL/min flow rate. FLAG-GST-DRD1 (337–446) or FLAG-GST-DRD1 (337–446, P445V) was captured on the measuring cell, and then one of eluate was injected. After elution, the chip surface was regenerated by 50 mM NaOH treatment for 30 sec.

Single amino acid substitution was introduced into each H444, P445 or T446 residue of FLAG-GST-DRD1 (337–446) by using PrimeSTAR Mutagenesis Kit (Takara Bio), and 16 FLAG-GST-DRD1 C terminus mutants were produced. Seventeen fusion proteins were cell-free synthesized, including wild type D1CE sequence and 16 D1CE mutants, and crude translation reaction mixture was subjected to Biacore assay as analyte. In each cycle, Ra62 antibody was captured at 2,000 RU at 5 μL / min. One of fusion proteins was injected at 10 μL/min for 180 sec. After 300 sec of dissociation time, the chip surface was regenerated by injecting protein G-regeneration solution for 30 sec at 10 μL/min.

### Protein purification

Purification of CP5-tagged soluble protein by peptide elution was conducted as follows. CP5 tagged protein was synthesized by wheat germ cell-free synthesis in 6 mL scale, and mixed with 100 μL of Ra62 antibody Sepharose equilibrated with HBS in advance. The mixture was mixed by rotation for 1 h at 4°C. The resin was transferred to micro spin column (GE Healthcare), and washed 5 times with 500 μL of HBS. 100 μL of elution buffer containing 150 μM of D1CE peptide in HBS was added to the resin. After 5-min incubation, the elution fraction was collected by centrifugation. Elution was repeated three times. Finally, the resin was mixed with 150 μL of SDS-PAGE sample buffer and incubated for 30 min at room temperature to analyze protein not eluted by peptide treatment. All fractions were subjected to SDS-PAGE and CBB staining.

GPCRs were cell-free synthesized using dialysis cup. 500 μL of cell-free protein synthesis reaction mixture, containing 125 μL of WEPRO7240 wheat germ extract, 125 μL of mRNA, 40 μg of creatine kinase and 5 mg of asoletin liposome, was put into a dialysis cup (10-kDa MWCO, Thermo Fisher Scientific) and immersed in 3.5 mL of substrate solution (SUB-AMIX SGC). The reaction was incubated at 22°C for 24 h. Then, the 500 μL translation reaction mixture was mixed with 2 mL of solubilization solution (150 mM NaHCO_3_, 750mM NaCl, 2% (w/v) n-dodecyl-β-D-maltopyranoside (DDM), 0.4% cholesterol hemi-succinate (CHS), 1 mM dithiothreitol (DTT), 10% glycerol at final concentration). The mixture was sonicated for 1 min then rotated 1 h. After centrifugation at 20,000 × g and 4°C for 15 min, supernatant was collected as solubilized GPCR fraction, and mixed with 100 μL of Ra62 antibody Sepharose equilibrated with wash buffer (15 mM Hepes-NaOH, 750 mM NaCl, 2% glycerol, 0.1% DDM, 0.03% CHS, 0.5 mM DTT, pH 7.4) in advance. The slurry was mixed by rotation at 4°C for 3 h, then transferred to empty column (GE healthcare). The resin was washed by 500 μL of wash buffer for 5 times. CP5 tagged GPCRs were eluted by 100 μL of elution buffer (150 μM D1CE peptide in wash buffer) for 3 times. Resin was then suspended by SDS-PAGE sample buffer and incubated for 30 min. All fractions were subjected to SDS-PAGE and CBB staining.

Insect-cell expressed CHRM2-CP5 was expressed and purified as follows. The CHRM2-BRIL fusion construct was generated by synthetic DNA (Genescript). C-terminal CP5-tagged CHRM2-BRIL fusion protein was cloned into pfastbac1 (Invitrogen) baculovirus expression vector with the haemagglutinin (HA) signal sequence followed by the N-terminally FLAG tag. 3C protease digestion sites were inserted between CHRM2 and CP5 tag sequence, and HA signal sequence and CHRM2. BRIL was substituted for residues 221–376 in the CHRM2 intracellular loop 3. The constructs were expressed in Sf9 insect cells using the Bac to Bac baculovirus system (Invitrogen). Cells were infected at a density of 3 to 4 × 10^6^ cells per ml and were grown for 48 h at 27°C. Cells were harvested by centrifugation and stored at −80°C.

Sf9 cell were lysed by osmotic shock in the presence of 10 uM QNB (Sigma-Aldrich). Receptor was extracted from cells using a Dounce homogenizer with a buffer of 30 mM HEPES-NaOH pH 7.5, 0.75 M NaCl, 5 mM imidazole, 1% (w/v) DDM, 0.2% Sodium cholate (Dojindo), 0.3% CHS, 1 mg/ml iodoacetamide (Dojindo) and Complete Protease inhibitor (Roche). The supernatant was isolated by ultracentrifugation for 30 min at 140,000g with a 50 kDa molecular weight cut-off concentrator. The suspension of membrane fraction was stored at –80°C. Solubilization buffer (150 mM NaHCO_3_, 250mM NaCl, 5% DDM, 1% CHS, 4 mM DTT, 10% glycerol at final concentration) was added to membrane fraction suspension. The mixture was sonicated for 10 min, rotated 2 h, and sonicated again for 10 min. After centrifugation at 20,000 × g and 4°C for 15 min, solubilized CHRM2-CP5 was collected. Five mL of solubilized CHRM2-CP5 sample was mixed with 15 mL of slurry containing 0.6 mL of Ra62 antibody Sepharose, 30 mM Hepes-NaOH, 250 mM NaCl, 4 mM DTT, pH 7.4. The mixture was rotated for 2h at 4°C. Sepharose resin was concentrated by centrifugation at 9,000 × g for 15 min, then transferred into an empty column (Bio-Rad). After washing by 10 bed volume of wash buffer (30 mM Hepes-NaOH, 250 mM NaCl, 0.1% DDM, 0.02% CHS, 10% glycerol, 4 mM DTT, pH 7.4), CHRM2-CP5 was eluted by adding elution buffer (150 μM D1CE peptide, 30 mM Hepes-NaOH, 250 mM NaCl, 0.1% DDM, 0.02% CHS, 10% glycerol, 4 mM DTT, pH 7.4). Finally, resin was suspended to buffer and the portion was transferred and mixed with ×3 SDS-PAGE sample buffer. All fractions were subjected to SDS-PAGE and CBB staining.

### Enzyme assays

Deubiquitination activity of CYLD-CP5 was evaluated as below. One hundred nM of purified CYLD-CP5 or Venus-CP5 was mixed with 1 μM recombinant M1-linked tetra ubiquitin (R&D Systems) in 12 μL of reaction mixture containing 50 mM Tris-HCl (pH 7.5), 5 mM DTT. The reaction mixtures were incubated at 30°C for 3 hours, then the reaction was terminated by boiling in SDS sample buffer. Degraded ubiquitin chain was visualized by SDS-PAGE and SYPRO Ruby Protein Gel Stain (Thermo Fisher Scientific).

In vitro self-ubiquitination assay of MARCH3 was conducted as follows. Five μL of purified MARCH3-CP5 or Venus-CP5 was mixed with 4 μM recombinant HA-ubiquitin (Boston Biochem) in 20 mM Tris-HCl (pH 7.5), 200 μM DTT, 5 mM MgCl2, 3 mM ATP, 40 nM recombinant E1 (Boston Biochem) and 300 nM recombinant UbcH6 (Enzo Life Sciences). The reaction mixture was incubated at 30°C for 3 hours. The reaction mixture was boiled in SDS sample buffer and subjected to SDS-PAGE followed by immunoblot analysis using HRP conjugated anti-HA antibody (Roche Life Science).

## Results

### Ra62 antibody recognized five C-terminal residues of DRD1 including its terminus carboxyl group

**Fig 1:**
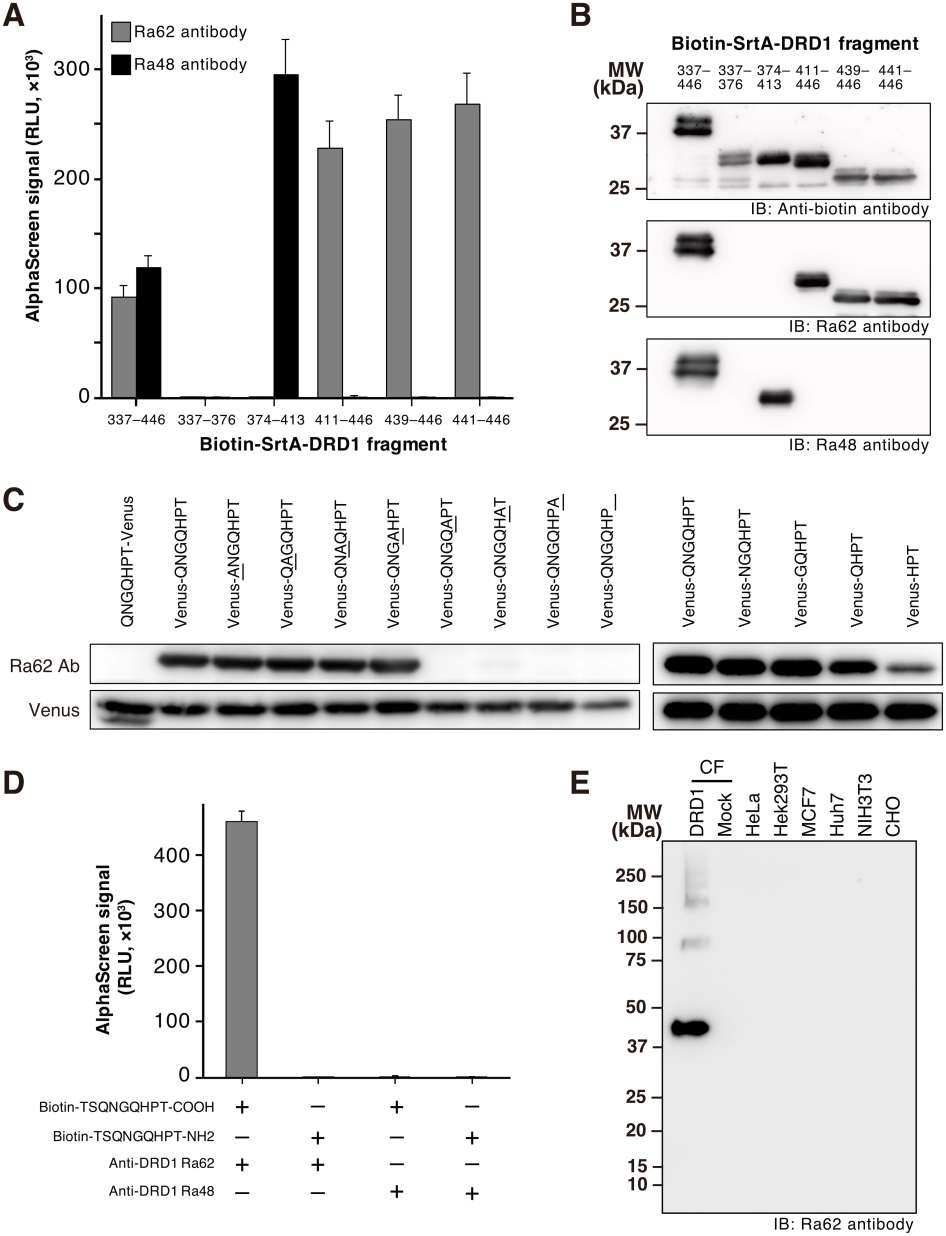
Determination of minimum epitope sequence of Ra62 antibody. (A,B) Narrowing epitope region of Ra62 antibody by AlphaScreen. Biotin-SrtA-DRD1 C-terminal fragments were cell-free synthesized. Interactions between DRD1 fragments and Ra62 or Ra48 antibodies were evaluated by AlphaScreen (panel A) and Western blotting (panel B). Error bars in panel A indicate standard deviation (n = 4). Synthesis of biotinylated fusion protein was visualized by using anti-biotin antibody (panel B). (C) Ra62 antibody recognized 5 residues of DRD1 at its C-terminus. C-terminal DRD1 7-residue fragment was fused with Venus protein [26], and alanine mutation and deletion were introduced into it. Mutant proteins were synthesized by wheat cell-free system, and subjected to Western blotting with Ra62 antibody and anti-rabbit IgG-HRP as primary antibody. Synthesis of Venus protein was visualized by using anti-GFP antibody. (D) Ra62 antibody recognized terminus carboxyl group in the epitope peptide. Interaction between antibody and biotinylated peptide was detected using AlphaScreen. (E) Specificity of Ra62 antibody. Extracts from wheat germ or cultured cells were subjected to Western blotting using Ra62 antibody as primary antibody. Cell-free synthesized DRD1 was applied as positive control recognized by Ra62 antibody. CF, reaction mixture of cell-free synthesis containing wheat germ extract.

In our previous report, we found that Ra62 antibody binds to C-terminal region of DRD1 [21]. In this study, we attempted to narrow down the epitope sequence of Ra62 using the same strategy as when we determined the epitope sequence of DRD1 recognized by Ra48 antibody [17]. DRD1 C-terminus region was separated into 4 fragments (337–446, 337–376, 374–413, 411–446), and each fragment was synthesized as biotin-SrtA fusion protein. Interactions between each DRD1 fragment and Ra62 antibody were monitored by AlphaScreen. Both Ra48 antibody and Ra62 antibody bound to C-terminus region of DRD1 (337–446), however, epitope of these antibodies were different (Fig 1A). Ra48 antibody bound to DRD1 374–413, whereas Ra62 bound to 411–446. We narrowed down the epitope region of Ra62 antibody, and found that it also bound to the shorter fragments 439–446 and 441–446. Western blotting was also conducted to confirm the AlphaScreen result (Fig 1B). Anti-biotin antibody detected all of proteins applied. Ra48 antibody detected 337–446 and 374–413. Ra62 antibody detected 337–446, 411–446, 439–446 and 441–446. The results well coincided with those of AlphaScreen, except for the signal intensity of 337–446. 337–446 fragment was strongly detected by both Ra48 and Ra62 antibodies in Western blotting, on the other hand, the AlphaScreen signals of 337–446 fragment detected by both Ra48 and Ra62 antibodies were half of ones of other shorter fragments.

To determine the minimum epitope sequence of Ra62 antibody, mutated DRD1 7-amino-acid fragment (441–446) was fused with Venus fluorescent protein. The fusion protein was synthesized by cell-free system, and then applied to Western blotting using Ra62 antibody as primary antibody (Fig 1C left panel). The results showed that Ra62 antibody did not recognized Venus protein with the 7-amino-acid fragment fused at the N-terminus (QNGQHPT-Venus). On the other hand, those fused at the C-terminus (Venus-QNGQHPT) was detected by Ra62 antibody. Next, we conducted alanine mutagenesis to the seven epitope amino acids. Alanine mutation was introduced into each position one by one in the 7-amino-acids fragment, and the interaction with Ra62 antibody was detected and evaluated by Western blotting. The result showed that Ra62 antibody did not bind to the mutant protein when any of last 3 amino acids was substituted with alanine residue. In addition, no signal was detected when without last threonine residue (Venus-QNGQHP_). These results indicate the importance of C-terminal 3 amino acids in recognition of Ra62 antibody. To confirm whether other residues contribute to Ra62 antibody binding, we made deletion mutants of the 7-amino-acid sequence (Fig 1C right panel). Deletion of 2 amino acids at the N-terminus of 7-amino-acid sequence did not affect the binding at all (Venus-GQHPT). However, binding signal of Ra62 antibody to Venus-QHPT and Venus-HPT was detected but obviously attenuated. These results demonstrate that HPT sequence was essential for Ra62 binding, and upstream GQ reinforced the binding.

Results shown in Fig 1C suggest the possibility that Ra62 antibody not only recognizes the epitope GQHPT residues but the carboxyl group of threonine as well, because Ra62 antibody did not bind to QNGQHPT-Venus. To verify that, we prepared two N-terminal biotinylated peptides by chemical synthesis. One of them contains Ra62 antibody epitope sequence (biotin-TSQNGQHPT-COOH) and the other one has the same peptide sequence but its terminal carboxyl group was substituted by an amide group (biotin-TSQNGQHPT-NH_2_). Interaction of Ra62 antibody with the peptides was examined by AlphaScreen. Fig 1D showed that Ra62 antibody strongly bound to the peptide with carboxyl group. Hhowever, it did not recognize the one with amide-group terminal. Ra48 antibody, a negative control, reacted with neither of the two peptides. This experiment confirmed that Ra62 recognized not only amino acid sequence GQHPT but also the terminus carboxyl group of threonine. Here, we name this GQHPT-COOH sequence DRD1 C-terminus epitope (D1CE).

We also tried to examine the specific recognition of Ra62 antibody. Reactivity of Ra62 antibody to the endogenous proteins in cell lysates including HeLa, HEK293T, MCF7, Huh7, NIH3T3, CHO cells. Cell-free synthesized DRD1 and mock reaction mixture of wheat cell-free system were also subjected. Western blotting with Ra62 antibody did not detect any intense bands in these cell lysates except cell-free synthesized DRD1 (Fig 1E). This result demonstrated that Ra62 recognizes no proteins in these major mammalian cultured cells.

### Development of CP5 tag

**Fig 2:**
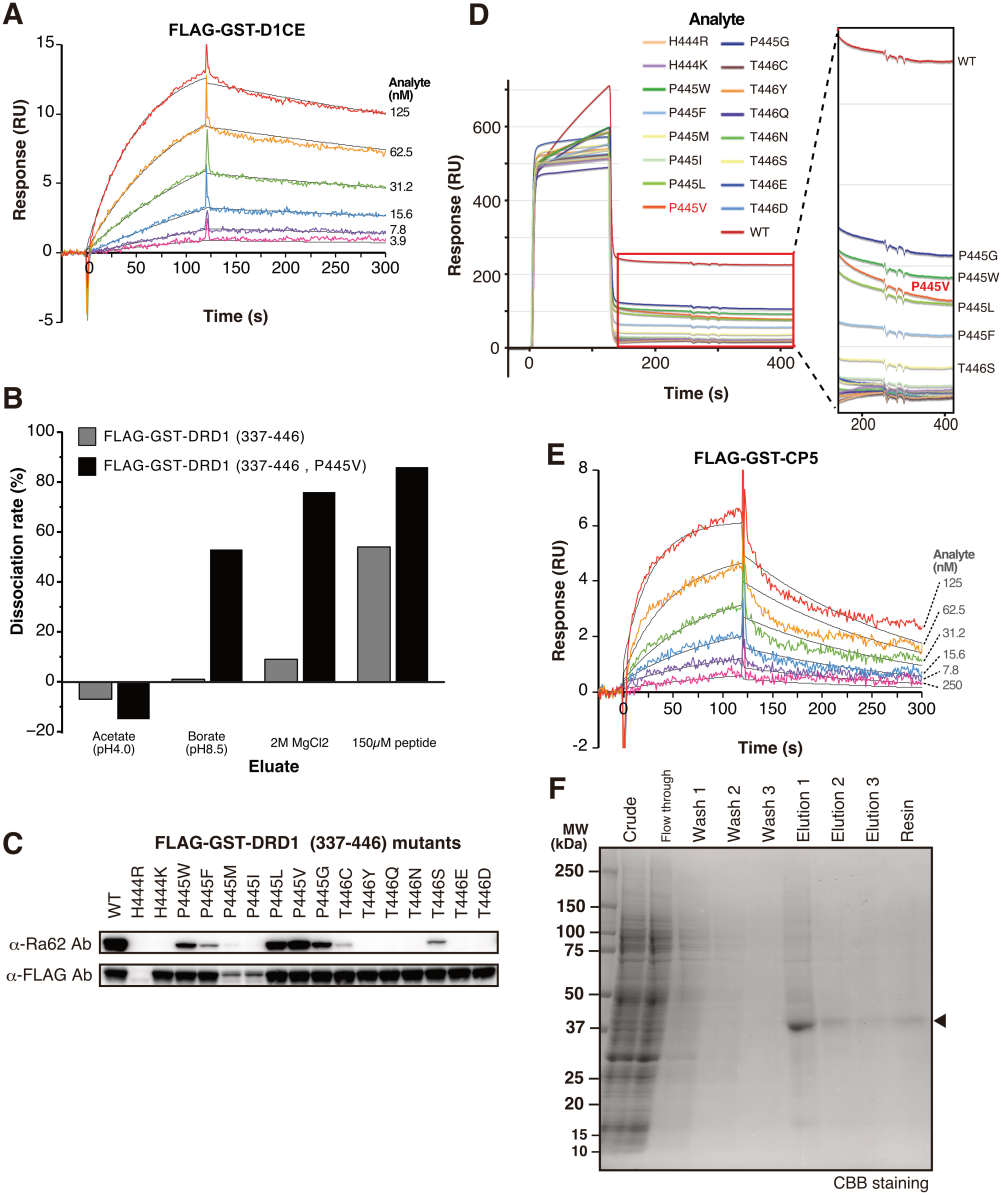
Development of CP5 tag. (A) Kinetics assay of D1CE epitope and Ra62 antibody. Ra62 antibody was captured on a protein G-immobilized sensorchip at 200 RU. Purified FLAG-GST-D1CE protein was then injected for 120 sec as analyte. Black lines represent a global fit of a 1:1 interaction model to each kinetic data set. (B) Elution scouting. Ra62 antibody was covalently immobilized on a sensorchip. In each cycle, FLAG-GST-DRD1 (337–446) or FLAG-GST-DRD1 (337–446, P445V) was injected and then an eluate was injected. Sensorgram of elution scouting is shown in S2 Fig. Dissociation rate is calculated by dividing binding response at 160 sec after eluate injection (S2 Fig, filled arrowhead) by one at 10 sec before eluate injection (S2 Fig, blank arrowhead). (C) D1CE mutants. Three residues on the C-terminus of FLAG-GST-DRD1 (337–446) were substituted one by one by similar amino acids. Their expression (anti-FLAG, lower panel) and Ra62 antibody reactivity (upper panel) were examined by Western blotting. (D) Binding assay of D1CE mutants. FLAG-GST-DRD1 (337–446) and mutants were injected to Ra62 antibody-immobilized sensorchip. Black box shows dissociation curves, in which difference in the dissociation rate was emphasized by stretching Y axis and shrinking X axis. (E) Kinetics assay of CP5 tag. Ra62 antibody was captured on a protein G-immobilized sensorchip at 200 RU. Purified FLAG-GST-CP5 protein was injected to Ra62 captured sensorchip for 120 sec as analyte. (F) Purification of CP5 tagged protein using CP5 system. Cell-free synthesis reaction mixture of FLAG-GST-DRD1 (337–446, P445V) protein was mixed with Ra62 antibody Sepharose. After washing, captured protein was eluted by D1CE epitope peptide. Purified protein was visualized by SDS-PAGE and CBB staining. Arrowhead indicates position of FLAG-GST-DRD1 (337–446, P445V).

We determined the affinity between D1CE sequence and Ra62 antibody using SPR. FLAG-GST-D1CE was cell-free synthesized, GST purified, and used as analyte. Kinetic analysis yielded a K_D_ value = 7.47 × 10^−9^ M (K_a_ = 1.43 × 10^5^ 1/Ms, K_d_ = 1.06 × 10^−3^ 1/s) (Fig 2A). We also examined kinetics assay using FLAG-GST-DRD1 fragment 337–446, 411–446, 439–446 and 441–446 (S1A–S1D Fig). Existence of additional sequences at the upstream of D1CE tag did not show a large difference in their kinetic parameters (S1E Fig).

Next, we examined the effects of several elution conditions on the affinity using SPR. We tried various eluates such as weak acid (acetate buffer pH 4.0), weak alkaline (borate buffer pH 8.5) and high concentration of divalent ion (2M MgCl_2_). The result showed that captured D1CE fusion proteins failed to be released efficiently under these elution conditions (Fig 2B and S2A Fig). Among the eluates tested, 150 mM D1CE peptide (TSQNGQHPT) eluted the captured analyte to some extent, however, half of the captured protein remained. To maximize the elution efficiency, we attempted to adjust the affinity between Ra62 and D1CE sequences using amino acid substitution.

To attenuate the affinity, we focused on the last 3 residues of D1CE sequence, HPT, which were critical for Ra62 recognition. Single amino acid substitution was introduced into these 3 residues in FLAG-GST-DRD1(337–446). Each residue was replaced with similar amino acids. R444 was substituted with positively charged hydrophilic amino acids. P445 was replaced with hydrophobic amino acids. T446 was substituted with uncharged or negatively charged hydrophilic amino acids. Eventually 16 mutant constructs were prepared and cell-free synthesized. Western blotting using anti-FLAG antibody showed that synthesis of H444R mutant was failed, and P445M and P445I mutations decreased protein production (Fig 2C). Other mutations showed no effect on protein synthesis. Among them, Ra62 antibody bound to 8 mutated epitopes. Immunoblot signal of these mutants was weaker than that of wild type. To analyze detailed mode of binding, we performed SPR assay. Ra62 antibody was captured on a sensorchip, these mutants were injected, and association and dissociation of them were investigated (Fig 2D). Besides wildtype, 6 mutants bound to Ra62. We noticed that dissociation rate of P445V was slightly faster than wildtype and other mutants (Fig 2D, black box). To confirm that P445V mutation accelerates dissociation, we determined kinetics of binding between Ra62 antibody and P445V mutant. SPR assay revealed that K_D_ value between Ra62 antibody and P445V mutant was 4.74 × 10^−8^ M (Fig 2E). Association rate of P445V mutant was similar to that of wildtype D1CE (K_a_ = 1.21 × 10^5^ 1/Ms), whereas dissociation rate was increased significantly (K_d_ = 5.72 × 10^−3^ 1/s). Next, elution efficacy of P445V mutant was evaluated in the same way as wildtype (Fig 2B and S2B Fig). Acid condition did not release FLAG-GST-DRD1 (337–446, P445V) captured by Ra62 antibody. On the other hand, elution by alkaline, Mg^2+^ and peptide was improved drastically compared with FLAG-GST-DRD1 (337–446). It is notable that more than 80% of captured protein was released by 150 mM D1CE peptide. We named this modified D1CE sequence (GQHVT) CP5 (C-terminus purification tag with 5 residues).

**Fig 3:**
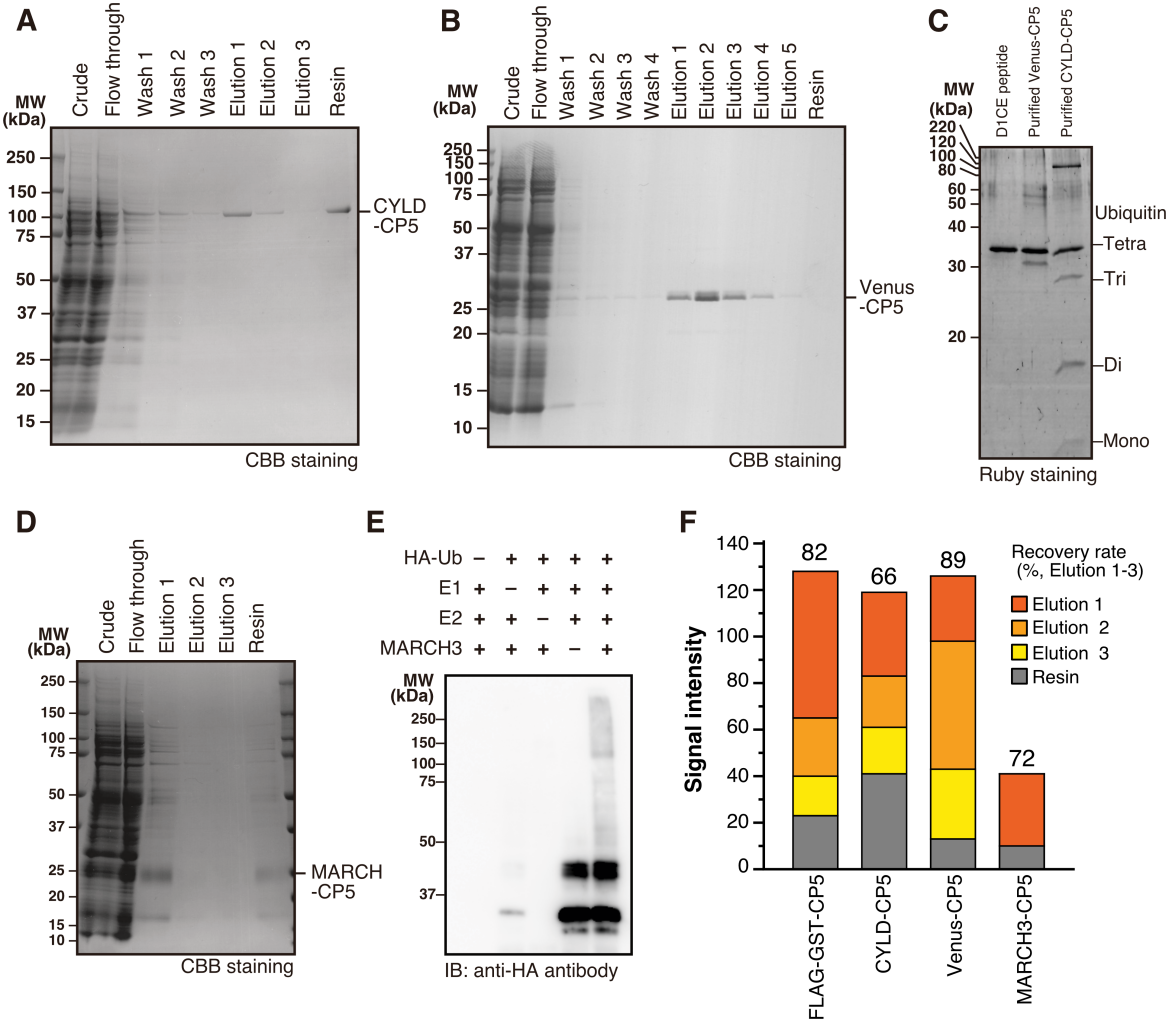
Purification of soluble proteins by CP5 system. (A) Purification of CYLD-CP5. Cell-free synthesized CYLD-CP5 was purified by CP5 system. Each fraction was subjected to SDS-PAGE and CBB staining. (B) Purification of control Venus-CP5 by CP5 system. (C) DUB assay. Linear tetra ubiquitin chain was treated with purified CYLD-CP5 and Venus-CP5. Degradation of products was visualized by SDS-PAGE and Ruby staining. (D) Purification of cell-free synthesized MARCH3-CP5 by CP5 system. Each fraction was subjected to SDS-PAGE and CBB staining. (E) Self-ubiquitination assay of purified MARCH3-CP5. Purified MARCH3-CP5 was mixed with E1, E2 (UbcH6), and HA-ubiquitin. Reaction mixture was applied to Western blotting. Ubiquitination was detected by HRP conjugated anti-HA antibody. (F) Recovery rate of soluble proteins purified by CP5 system. Signal intensity of each band in CBB staining images was measured by using ImageJ software. After background noise level was subtracted, signal intensity of CP5 tagged protein was plotted. Figures on the bar graph indicate recovery rate calculated by dividing recovered target proteins (elution 1 to 3) by total captured proteins (elution 1 to 3 and resin).

Using Ra62 antibody and 2 peptides with different affinity, we developed a protein purification method, CP5 system. CP5 tag is fused to the C-terminus of a target protein, and Ra62 antibody captures CP5-tagged protein. Finally, free peptide containing D1CE sequence, a strong and competitor that has 10 times higher affinity to CP5, effectively elutes the captured protein. To investigate the performance, CP5 tagged protein was purified by CP5 system. For stable capture, Ra62 antibody was immobilized on Sepharose beads using amine coupling. Cell-free synthesized FLAG-GST-DRD1 (337–446, P445V) protein was mixed with Ra62 antibody Sepharose. After washing, the captured protein was successfully eluded by 150 mM D1CE peptide treatment (Fig 2F). Among 3 repeated elution runs, most protein was sharply eluted in the first fraction as a major band. Although small amount of protein was detected in the SDS-eluted resin fraction, 82% of protein was recovered by peptide elution (Fig 3F). The recovery rate showed good agreement with the elution scouting test in Biacore (Fig 2B).

### Purification of enzymes by CP5 system

To see if CP5 system is applicable to enzyme preparation, we tried to purify soluble enzyme and evaluated its enzymatic activity. First, CP5 tag was added to the C-terminus of CYLD. CYLD is a deubiquitinating enzyme (DUB), which selectively degrades K63- and M1-linked ubiquitin chain and negatively regulates NF-κB signal pathway [27]. Biochemical DUB assay requires highly purified and catalytically active CYLD to remove endogenous DUB enzymes, and thus was used to evaluate CP5-system-purified CYLD activity. CYLD-CP5 was cell-free synthesized and purified using CP5 system (Fig 3A). CYLD is much larger (107 kDa) than GST; however, it was well purified with high purity. Portion of CYLD-CP5 was remained on the resin after elution, and the recovery rate was 66% (Fig 3F). Venus fluorescent protein was also fused with CP5 tag, cell-free synthesized and purified as negative control (Fig 3B). Purified CYLD-CP5 successfully deubiquitinated M1-linked tetra ubiquitin chain, and produced monomer, dimer, and trimer ubiquitin (Fig 3C), demonstrating that CP5 system has no negative effects on CYLD activity. On the other hand, D1CE peptide or purified Venus-CP5 did not degrade tetra ubiquitin chain. These data indicate that CP5 system removed all endogenous DUB enzymes well.

**Fig 4:**
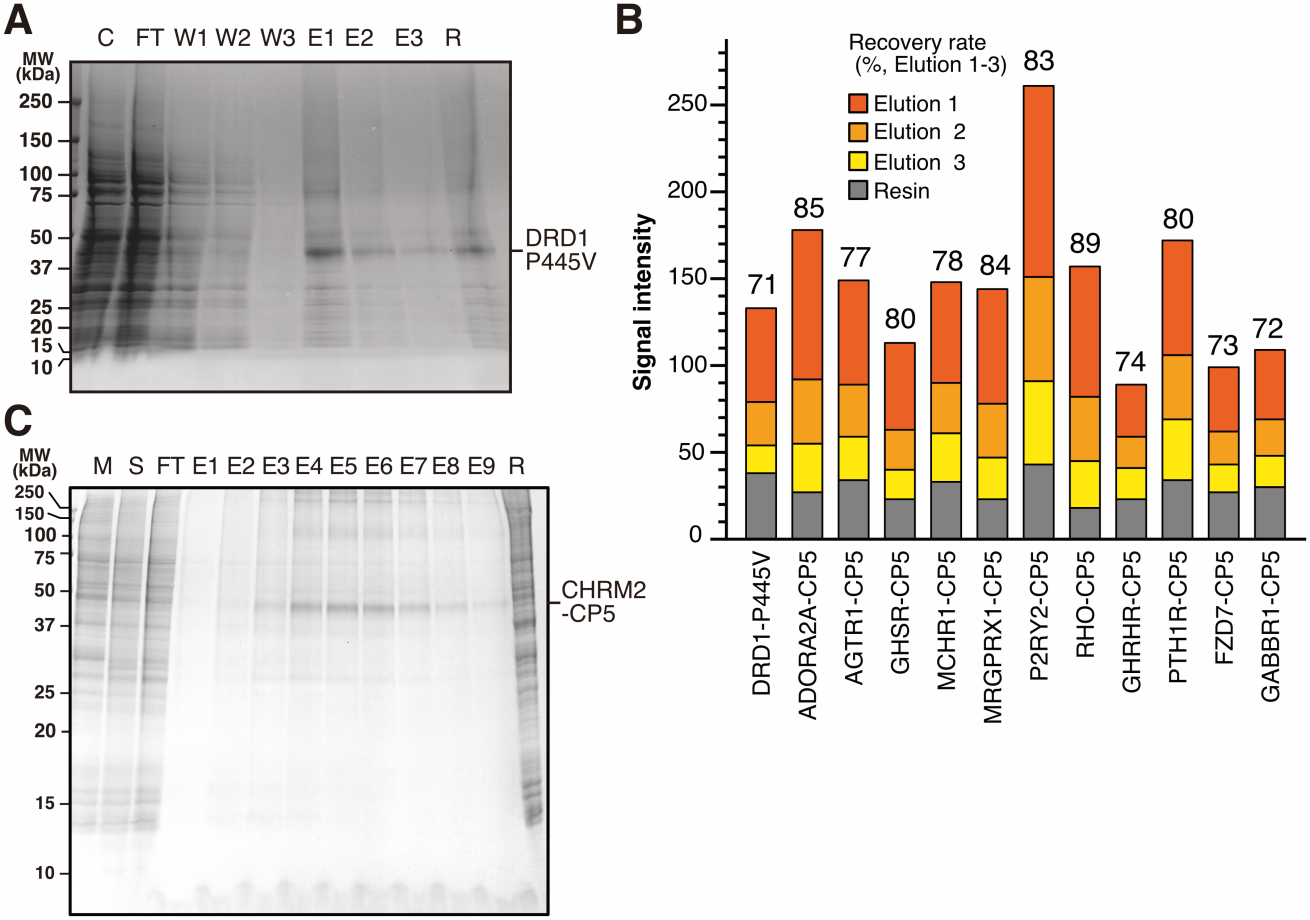
Purification of GPCRs by CP5 tag. (A) Purification of DRD1 P445V. Cell-free synthesized DRD1 P445V proteoliposome was solubilized by DDM, and was applied to CP5 system. Each fraction was analyzed by SDS-PAGE and CBB staining. C, crude; FT, flow through; W1-3, wash 1 to 3; E1-3, elution 1 to 3; R, resin. (B) Recovery rate of GPCRs purified by CP5 system. Figures on the bar graph indicate recovery rate calculated by dividing recovered target proteins (elution 1 to 3) by total captured proteins (elution 1 to 3 and resin). (C) Purification of CHRM2-CP5 expressed by baculovirus-insect cell system. CP5 tagged CHRM2 was expressed in SF9 cells. Membrane fraction was isolated and solubilized by DDM, and solubilized CHRM2-CP5 was purified by CP5 system. Each fraction was applied to SDS-PAGE and CBB staining. M, cell membrane fraction; S, solubilized fraction; FT, flow through; E1-9, elution 1 to 9; R, resin.

Another enzyme to verify the applicability of CP5 tag purification system is E3 ubiquitin ligase MARCH3 (Fig 3D). Limited amount of MARCH3-CP5 was captured compared with other proteins examined in this study (Figs 3F and 4B). 72% of captured MARCH3-CP5 was eluted in the first fraction of elution. Several extra bands were observed in the purified MARCH3-CP5 fraction. However, Western blotting using Ra62 antibody did not detect these extra bands except for 30-kDa MARCH3-CP5 (S3 Fig), implying that the extra bands were proteins interacted and co-precipitated with MARCH3 during the purification. Purified MARCH3-CP5 was subjected to self-ubiquitination assay. Only when all the components required for ubiquitination, including HA tagged ubiquitin, commercial E1, commercial E2, and CP5 purified MARCH3, were added to the reaction mixture, smeared ladder-shape poly-ubiquitin bands were observed (Fig 3E, right lane), suggesting that CP5 purified MARCH3-CP5 and meanwhile retained its ubiquitin ligase activity.

### Purification of GPCRs by CP5 system

To demonstrate applicability of CP5 system to integral membrane protein purification, we purified several GPCRs using CP5 system, as representative integral membrane proteins. First, we conducted purification of DRD1, the antigen of Ra62 antibody and origin of D1CE sequence. P445V mutation was introduced into DRD1 to make its C-terminal sequence same as CP5 tag. DRD1-P445V was synthesized in vitro with liposome, and DRD1-P445V liposome was solubilized by 2% DDM. After solubilization, DRD1 P445V was mixed with Ra62 antibody Sepharose to be captured on the resin. Followed by washing with buffer containing 0.1% DDM, captured DRD1 was eluted by 150 mM D1CE peptide. Similar to soluble proteins, DRD1 P445V was effectively eluted (Fig 4A). Although some part remained on the resin, 71% of captured DRD1 P445V protein was released by D1CE peptide, and majority was eluted in the first fraction.

Besides DRD1 P445V, we furthermore attempted to purify other 11 GPCRs using CP5 system. We inserted CP5 tag right before stop codon in these GPCRs without linker. Cell-free synthesized CP5 fusion GPCRs were solubilized and CP5 purified (S4A–S4K Fig). These GPCRs were well captured by the antibody and released by peptide. The recovery rate was from 72% to 89% (Fig 4B). Majority of captured proteins were eluted in the first elution fraction. GHRHR, FZD7 and GABBR1 showed relatively low capture amount and recovery rate compared with other 8 GPCRs. Several extra bands were observed in elution 1 fraction of DRD1 (Fig 4A), ADORA2A, P2RY2, RHO (S4A, S4F and S4G Fig). As to MARCH3, no such extra bands were detected using Ra62 in Western blotting (S3 Fig).

Not only cell-free synthesized membrane proteins, CP5 system also worked well for purification of CP5-tagged GPCR expressed on a cell membrane. CP5 tag was fused at the C-terminus of CHRM2, and the tagged protein was expressed by baculovirus-insect cultured cell system. Membrane fraction was isolated and CHRM2-CP5 was solubilized with solubilization buffer containing 4% DDM. The solution was diluted 4 times and mixed with Ra62 antibody Sepharose. After washing, the captured CHRM2-CP5 was eluted well by 150 mM D1CE peptide treatment (Fig 4C). CHRM2-CP5 was mainly eluted in fraction 4 to 7 sharply, and target protein was observed as a clear major band in each fraction.

## Discussion

Using Ra62 antibody and two peptide sequences, we developed CP5 protein purification system that has several biochemical advantages and unique characteristics. Previously we developed and reported AGIA tag [17]. Both D1CE tag and AGIA tag were developed using similar strategies, however, they have different structural and biochemical properties. One characteristic of CP5 system is the shortness of the tag sequence. D1CE/CP5 tags consist of 5 amino acid residues, and are much shorter than 9-residue AGIA tag. Until now, C-tag (Thermo Fisher Scientific) is known as the shortest tag, which recognizes 4-residue peptides fused at the C-terminus of a target protein. D1CE and CP5 tags are the next shortest ones. Molecular weight of D1CE and CP5 is only 538 and 540, respectively. Theoretically, such a small tag is hard to have an influence on chemical property of tagged proteins, such as conformation and enzyme activity. Another advantage we would like to address is the simplicity of CP5 tag construction. Using site-directed mutagenesis with inverse PCR, 15-base-long nucleotides coding CP5 tag can be inserted into any existing expression plasmid easily and quickly. On the other hand, we also need to discuss the potential risks of short CP5 tag. One common disadvantage of short tag is the exposure of a tag. Some proteins fold their terminal end inside with fused short tag to prevent interaction between capture antibody and the tag. Indeed, AlphaScreen signal between Ra62 antibody and DRD1 fragment (337–446) was significantly lower than shorter fragments (Fig 1A), however, signal intensity of 337–446 detected by Western blotting using Ra62 antibody showed same or higher than others (Fig 1B). The difference between AlphaScreen and Western blotting is conformation of the proteins. AlphaScreen detect interaction under the native state, whereas samples were denatured in Western blotting. Furthermore, Biacore experiment revealed that kinetics of 337–446 fragment and Ra62 antibody was not much different from the one of shorter fragments (S1 Fig). Taking together these results, we believe that the upstream sequence of D1CE have no effect on the affinity between Ra62 antibody and the epitope, however, possible conformation change of the longer fragment block the interaction between antibody and the short epitope. This problem can be resolved by inserting some flexible linker between target protein and a tag. Another possible risk is the specificity of capture antibody. Expression of DRD1 is found limited to central nervous system, but suppressed in other tissues and major cultured cells [17]. However we were concerned about the possibility of cross-reactivity of Ra62 antibody with other endogenous proteins because its epitope was only 5 residues. We searched RefSeq database (RefSeq release 79, released in November 2016) to confirm how many proteins have D1CE or CP5 tag sequence at their C terminus. Although there are lots of proteins containing GQHPT or QHPT sequences in the middle of their peptide sequences, however, no human protein was found to have these epitope sequences at the C terminal except for DRD1. Also, no human protein contained GQHVT-COOH or QHVT-COOH, either. Furthermore, Western blotting showed that Ra62 antibody did not react with endogenous proteins in the cell lysate from several mammalian cultured cell lines as well as wheat germ extract (Fig 1D). Several CP5-purified proteins contained endogenous contaminated proteins (Figs 3D and 4A, S4A, S4F and S4G Fig), and these contaminated proteins were not detected by Ra62 antibody (S3 Fig). Taken together with the result that extra-band patterns of each purified fraction were inconsistent, we speculate that those observed extra bands were endogenous wheat germ proteins, which interacted and co-precipitated with target proteins, neither Ra62 antibody nor Sepharose. Based on the above data, we conclude that Ra62 antibody specifically recognizes CP5-tagged proteins, and there is almost no risk for Ra62 antibody to cross-react with human endogenous proteins.

High performance of CP5 system is realized by both efficient capture and elution. Ra62 antibody quickly captures CP5-tagged target protein (Fig 2E), and captured CP5-tagged protein was eluted by competing with D1CE peptide. By taking the difference of the affinity between D1CE and CP5 (S1E Fig), sharp elution was achieved (Fig 2F). P445V mutation in D1CE tag significantly increased the dissociation from Ra62 antibody. E2D mutation in AGIA tag did not show much difference in kinetics compared with wild type tag sequence [17]. The contrast of affinity between D1CE and CP5 tag is the advantage of CP5 system that AGIA tag system does not have. Competitive elution of peptide provides both high elution efficacy and moderate condition. High concentration of di-cation would be an alternative eluate (Fig 2B), with which cost-effective purification could be realized under relatively mild condition. The high performance and mildness of CP5 system guarantee the enzyme preparation with sufficient quality for biochemical studies, such as functional assay, structural analysis, or drug screening.

In this report, we demonstrated that CP5 system successfully purified integral membrane proteins with good purity and recovery rate (71–89%) (Fig 4 and S4 Fig). Integral membrane proteins, including GPCRs, are gaining popularity as one of the most important drug targets, and many structural analyses of integral membrane proteins have been performed for research and development of medicines. Structural analysis of integral membrane protein requires large amount of, highly purified, and active protein samples, and its purification remains a critical bottleneck of sample preparation for several technical reasons. Our results demonstrate that CP5 system provides solutions for that. First, high performance capture antibody is required in integral membrane protein purification, because its capture is conducted under severe condition coexistent with large amount of detergents, lipids, and other membrane proteins. Capture of CP5 system is conducted by Ra62 antibody, which is a rabbit monoclonal antibody with both high specificity and rigidity. Indeed, Ra62 successfully captured CP5-tagged CHRM2 in solubilized SF9 membrane fraction containing 1.25% DDM (Fig 4C). Second, physiological condition should be maintained during purification to avoid damage to a target integral membrane protein, which has complicated and unstable structure. As described in the former paragraph, competitive peptide elution of CP5 provides mild purification condition, and it is also advantageous in maintaining the structure of membrane proteins as well as enzymes. We also should note that integration of CP5 system with other affinity purification strategies should be considered to furthermore improve the purity. High elution efficiency of CP5 system is advantageous to construct dual- or multi-tagging purification. We believe that CP5 system provides a new promising option for sample preparation of biochemical and structural analysis of integral membrane proteins.

## Supporting information

S1 Fig Kinetics assay between Ra62 antibody and DRD1 fragments. (A-D) Kinetics assay of Ra62 antibody and FLAG-GST-DRD1 fragments. Ra62 antibody was captured on a protein G-immobilized sensorchip at 200 RU. A purified FLAG-GST-DRD1 fragment protein was then injected for 120 sec as analyte. Black lines represent a global fit of a 1:1 interaction model to each kinetic data set. (E) Kinetic parameters between Ra62 antibody and DRD1 fragments.
(TIFF)

S2 Fig Elution scouting of D1CE/ CP5 tagged protein captured by Ra62 antibody. Ra62 antibody was covalently immobilized on a sensorchip at 6,000 RU. In each cycle, FLAG-GST-DRD1 (337–446) (panel A) or FLAG-GST-DRD1 (337–446, P445V) (panel B) was injected and then an eluate was injected.
(TIFF)

S3 Fig Western blotting of purified proteins by CP5 system. The first elution fraction of each protein was applied to SDS-PAGE and Western blotting. Ra62 antibody and anti-rabbit IgG-HRP were used as primary and secondary antibodies, respectively. Arrowheads indicate target proteins.
(TIF)

S4 Fig Purification of cell-free synthesized GPCRs by CP5 system. CP5 tag was fused at the C terminus of 11 GPCRs, respectively. Name of each GPCR is shown under the panel. CP5 tagged GPCRs were synthesized by wheat cell-free system as proteoliposome, solubilized by DDM, and purified by CP5 system. Each fraction was applied to SDS-PAGE and CBB staining. Arrowheads indicate band position of target proteins. C, crude; FT, flow through; E1-3, elution 1 to 3; R, resin.
(TIFF)
