# Supplementary figures and images for: CP5 system, for simple and highly efficient protein purification with a C-terminal designed mini tag
Source: PLoS One. 2017 May 25;12(5):e0178246. doi: 10.1371/journal.pone.0178246 (PMC5444806; doi:10.1371/journal.pone.0178246)

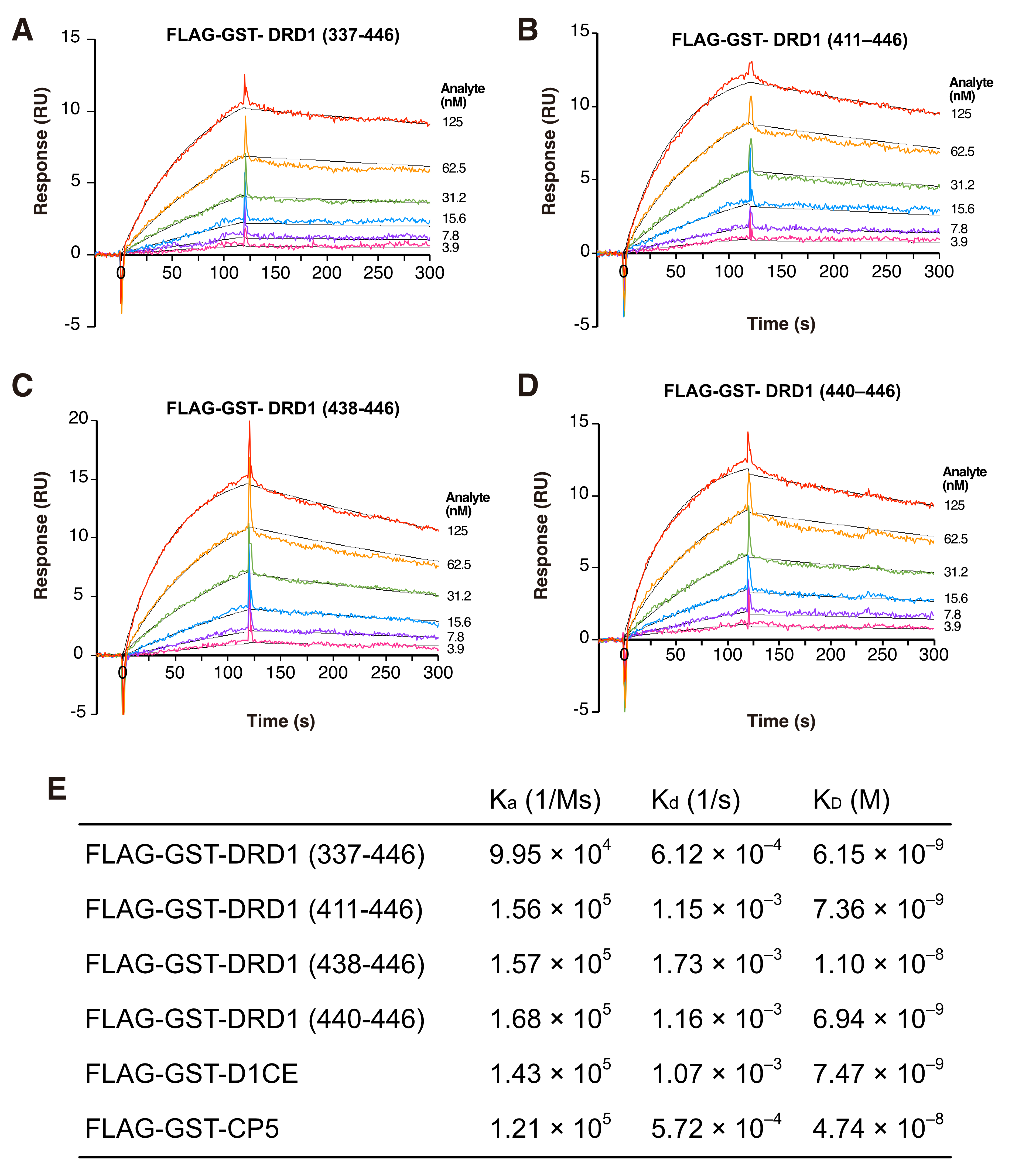

Supplement: S1 Fig — (A-D) Kinetics assay of Ra62 antibody and FLAG-GST-DRD1 fragments. Ra62 antibody was captured on a protein G-immobilized sensorchip at 200 RU. A purified FLAG-GST-DRD1 fragment protein was then injected for 120 sec as analyte. Black lines represent a global fit of a 1:1 interaction model to each kinetic data set. (E) Kinetic parameters between Ra62 antibody and DRD1 fragments. (TIFF) [file pone.0178246.s001.tiff]

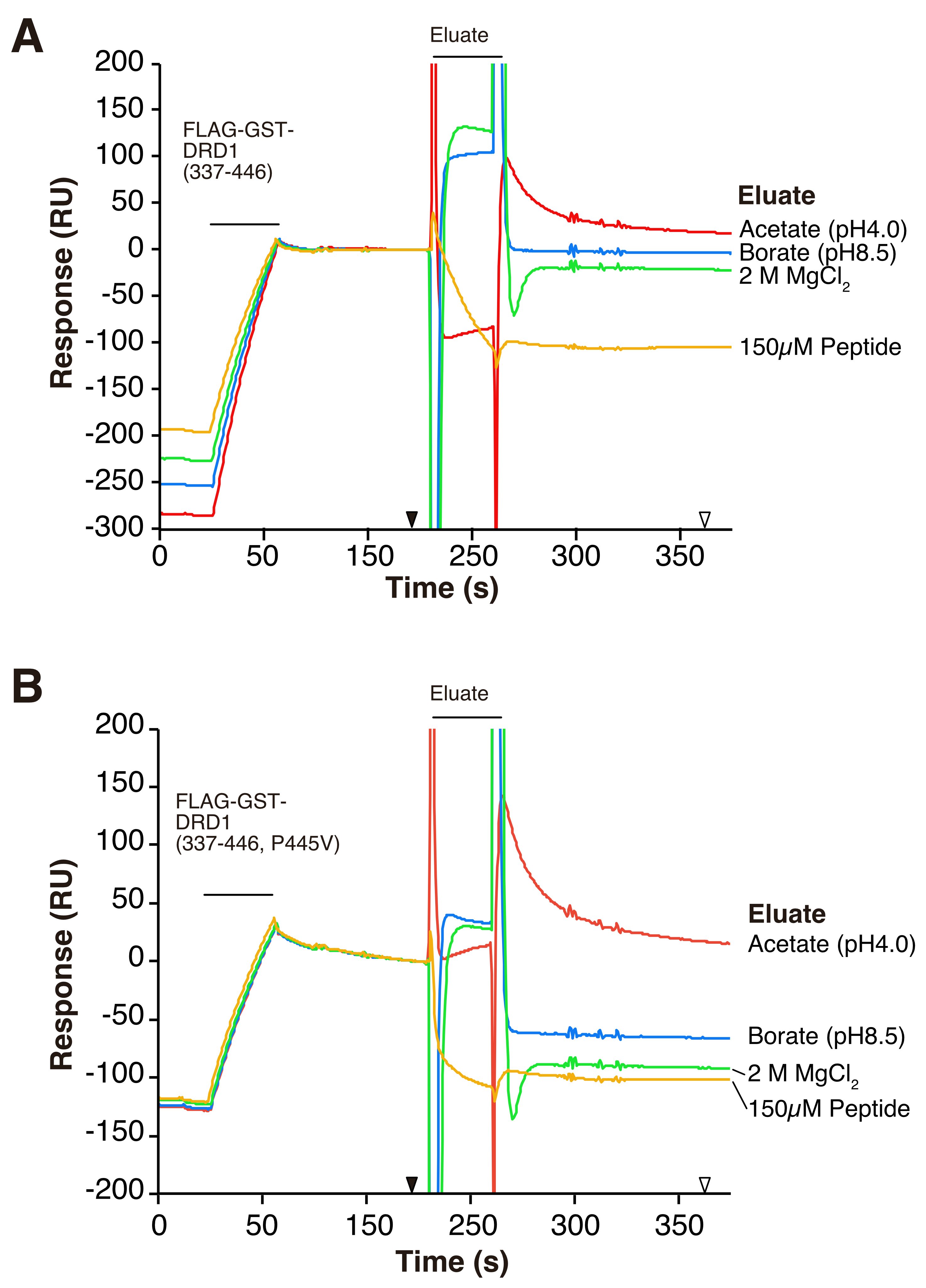

Supplement: S2 Fig — Ra62 antibody was covalently immobilized on a sensorchip at 6,000 RU. In each cycle, FLAG-GST-DRD1 (337–446) (panel A) or FLAG-GST-DRD1 (337–446, P445V) (panel B) was injected and then an eluate was injected. (TIFF) [file pone.0178246.s002.tiff]

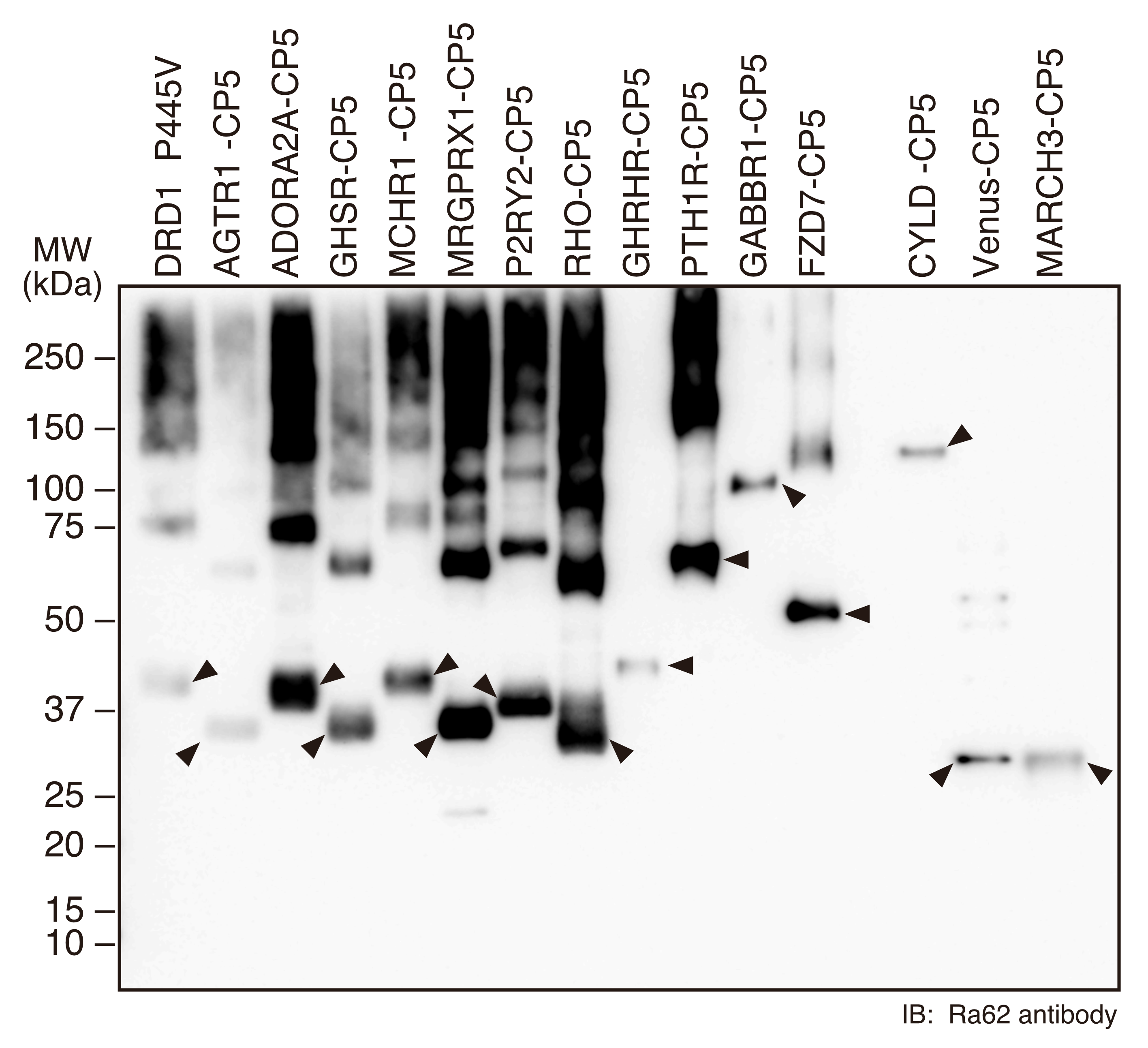

Supplement: S3 Fig — The first elution fraction of each protein was applied to SDS-PAGE and Western blotting. Ra62 antibody and anti-rabbit IgG-HRP were used as primary and secondary antibodies, respectively. Arrowheads indicate target proteins. (TIF) [file pone.0178246.s003.tif]

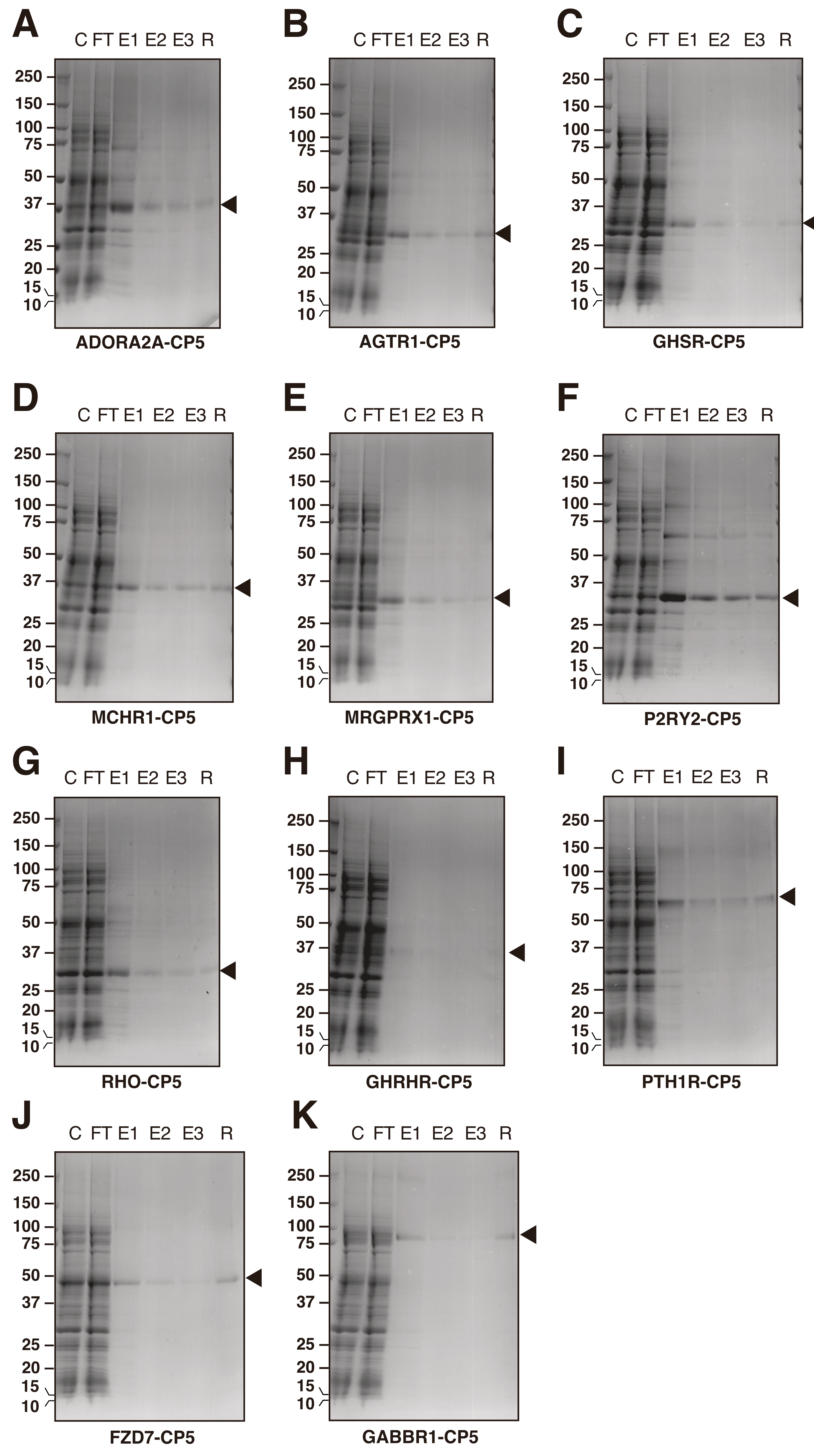

Supplement: S4 Fig — CP5 tag was fused at the C terminus of 11 GPCRs, respectively. Name of each GPCR is shown under the panel. CP5 tagged GPCRs were synthesized by wheat cell-free system as proteoliposome, solubilized by DDM, and purified by CP5 system. Each fraction was applied to SDS-PAGE and CBB staining. Arrowheads indicate band position of target proteins. C, crude; FT, flow through; E1-3, elution 1 to 3; R, resin. (TIFF) [file pone.0178246.s004.tiff]
